# Nutritional, Bioactive, and Volatile Characteristics of Two Types of *Sorbus domestica* Undervalued Fruit from Northeast of Iberian Peninsula, Spain

**DOI:** 10.3390/molecules29184321

**Published:** 2024-09-12

**Authors:** María Dolores Raigón Jiménez, María Dolores García-Martínez, Patricia Esteve Ciudad, Tamara Fukalova Fukalova

**Affiliations:** 1 Instituto de Conservación y Mejora de la Agrobiodiversidad Valenciana, Universitat Politècnica de Valencia, Camino de Vera s/n, 46022 Valencia, Spain; magarma8@upv.edu.es (M.D.G.-M.); pestevec@upv.edu.es (P.E.C.); 2 Laboratorio de Fitoquímica y Productos Biológicos, Facultad de Ciencias Químicas, Universidad Central del Ecuador, Avenue Universitaria, Quito 170521, Ecuador; tfukalova@uce.edu.ec

**Keywords:** biodiversity fruits, antioxidant, maturation stages, minerals, aromatic, proximal

## Abstract

The promotion of food from underutilized plants can help combat biodiversity loss, foster cultural preservation, and empower farmers in the face of market pressures and sustainable production conditions. The nutritional and aromatic characterization of two undervalued types of *Sorbus domestica* fruits, differentiated by their apple and pear shapes, has been carried out. Official Association of Analytical Communities methods have been used for proximate composition and mineral analysis determinations, and gas chromatography was used for the analysis of volatile components in three states of ripeness and compared with the aromas of fresh apple and quince jam. *S. domestica* fruits are a good source of K, Ca, Fe, and fiber and are an important source of antioxidants in the human diet. *S. domestica* fruits have proven to be very distinctive in the aromatic fraction. 1-hexanol, hexyl 1,3-octanediol, phenylacetaldehyde, nonanal, hexanal, and α-farnesene are the most potent odor compounds in the overripening stage of the fruits. The aroma profiles of immature *S. domestica* fruits were dominated by aldehydes, while in the overripe stage, the fruit accumulated abundant esters, alcohols, and sesquiterpenoids. *S. domestica* fruits could be introduced as an alternative to seasonal fruit consumption and could generate sustainable production and consumption alternatives while recovering cultural and food heritage.

## 1. Introduction

The plant kingdom in its constant evolution has been the sustenance of biologically active constituents of food and raw materials indispensable for humanity [1]. In this sense, fruit species have been a vital source of food and medicinal resources. Plant species have maintained a close relationship with various aspects of the social, economic, and cultural lives of ancient peoples. In this close relationship, each people and each culture has acquired knowledge and know-how about its environment, which together form part of an incalculable intangible heritage of biodiversity [2].

In recent decades, crops have been standardized and diets homogenized, resulting in only 94 species accounting for 90% of the global food supply [3]. Dietary homogenization has led to an increased global supply of energy-dense foods. But global diets are poorer and characterized by a high consumption of refined carbohydrates, added sugars and fats, and an increase in animal-sourced foods, while legumes, vegetables, minor crops, and wild foods have become less relevant [4].

Environmental impacts in terms of greenhouse gas emissions, biodiversity loss, and water and soil degradation are closely related to the diet patterns of the world’s population [5]. There exists a strong connection and a clearly hegemonic role of the agri-food industry in shaping the dietary pattern of the population [6]. According to the latest scientific research [7,8], the adoption of a healthy dietary pattern will considerably reduce the footprints of the current diet (carbon, water, and territorial). 

The shift to dietary patterns that are more diverse and sustainable includes greater consumption of fruits and vegetables [9] and underutilized plant species, which are crucial components of achieving global food security and promoting biodiversity conservation [10]. These often-overlooked plants encompass a vast array of species that have been traditionally consumed by various cultures but have faded into oblivion due to the dominance of a few staple crops in modern agriculture and food systems [11]. Underutilized crops often possess adaptive traits, such as drought tolerance or pest resistance, that can enhance the overall resilience of agroecosystems. Reintroducing these plants into agricultural landscapes can promote ecological stability and reduce the reliance on intensive agricultural practices that degrade soil health and contribute to biodiversity loss. Katoch (2020) [12] conducted a review on the importance of underutilized crops, examining the global distribution of these crops. In the Asian-Pacific area, they described the presence of eight legumes, five pseudocereals, three tubers, six vegetables, and six fruit trees, including *Vigna aconitifolia*, *Echinochloa colona*, *Ipomoea mammosa*, *Basella rubra*, and *Mimusops hexandra*, respectively. For the African continent, four legumes, one tuber, and six vegetables were described, including *Vigna subterranea*, *Plectranthus esculentus*, and *Solanum scabrum*, respectively. And in North and South America, four tubers and two types of fruits were described, including *Smallanthus sonchifolius* and *Byrsonima crassifolia*.

Furthermore, the promotion of underutilized plant species can foster cultural preservation and empowerment. Many of these plants hold significant cultural and historical importance for various regions, serving as symbols of identity and tradition [13]. By revitalizing the cultivation and consumption of these crops, we can not only safeguard traditional knowledge and practices but also empower local communities to assert control over their food systems and cultural heritage. Consequently, food sovereignty and social justice are promoted, ensuring that marginalized groups have access to culturally appropriate and nutritious foods. Underutilized plant species also offer economic opportunities for smallholder farmers and rural communities. Through value-added processing and marketing initiatives, these crops can generate income and employment opportunities along the value chain, contributing to poverty alleviation and rural development [14].

Among these undervalued fruit trees is *Sorbus domestica* L. It is a deciduous tree belonging to the Rosaceae family. It is native to Europe, Asia Minor, and the Caucasus. It has been cultivated since ancient times for its edible fruits and high-quality wood. Currently, this species is present in areas of southern and central Europe, with its presence being more notable around the Balkan Peninsula, in the Italian Peninsula, and occasionally in the Iberian Peninsula and France. Of particular note is the sporadic occurrence of some specimens in North Africa, the British Isles, and the Caucasus area [15]. The presence of *S. domestica* has decreased in some areas due to deforestation, habitat loss, and the implementation of other more commercial fruits in the markets. In fact, in some regions, such as Austria, Germany, and Spain, this species is endangered [15,16]. However, in recent decades, there has been a renewed interest in its cultivation due to its ecological value and its potential as a source of quality fruit and wood [17].

**Figure 1:**
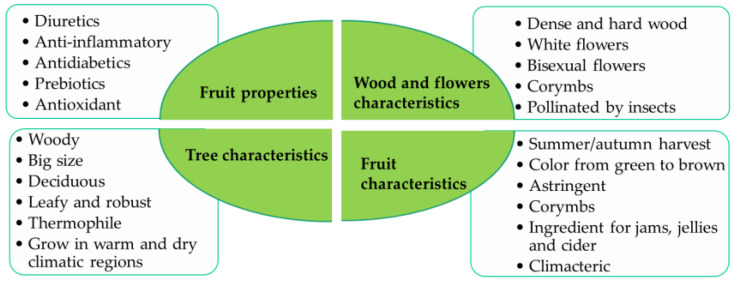
General characteristics of *Sorbus domestica*.

*S. domestica* is a valuable tree both for its practical uses and for its contribution to the environment. The main characteristics of this species are shown in Figure 1. Its cultivation and conservation can offer multiple benefits, from food products and durable materials to improving the biodiversity and ecological stability of the regions where it grows [18]. In some regions of Spain, it has been classified as a vulnerable species and is at risk of declining [16]. The fleshy *S. domestica* fruits with smooth skin differ in shape and size. Their color varies from greenish to reddish tones depending on their state of maturity [19]. The fruits of *S. domestica* L. are very astringent until they ripen due to rapid enzymatic fermentation, at which time they acquire a brown color and pleasant flavor. The fruits can be consumed in different forms such as jams or jellies [20]. There is also evidence that since Roman times, the fruits of *S. domestica* have been used to cure intestinal problems and as an additive for preserving apple cider [21]. They are also used in some areas to make distilled beverages [22,23]. In the community of Xanthi (Greece), the population uses this fruit, not only as food but also as a traditional astringent, antidiarrheal, and antidiabetic agent in the form of pulp [24].

There is little information on the nutritional composition of *S. domestica* fruits [25], and no data have been found that incorporate the nutritional composition, differentiating between the different morphologies of the fruit. Some research has been carried out on the chemical composition and nutritional value of *S. domestica* fruits [24,26,27,28,29,30,31]. Some research focuses on the presence and accumulation of some minerals [26,32], and others point to the fruits as a source of phenolic compounds [27,28,30,33] and the variation of the phenolic composition at different stages of maturity [29,30,31]. The aim of this work is to deepen the understanding of the nutritional, biochemical, and volatile properties of the fruit of *S. domestica*. This work evaluated the differences between the nutritional and biochemical properties of two types of *S. domestica* fruits, designated as apple and pear based on their characteristic shapes. For this purpose, a proximate analysis and quantification of biochemical components, in addition to volatile composition, were carried out. The proximate analysis of minerals, minor components, total polyphenols, and antioxidant activity by DPPH assay was carried out at the time of overripening, when the fruits were ready for consumption. The typification of the volatile composition of *S. domestica* was evaluated at three stages of ripening to determine its organoleptic characteristics related to aromas and flavors from its green state until consumption. This work aims to be a reference for promoting the inclusion of this fruit as a nutritional alternative to increase interest in nutrition and the consumption of foods beneficial to health, particularly to local and seasonal foods.

## 2. Results

### 2.1. Individual Weights, Proximal and Mineral Composition of S. domestica Fruits

The results of the average individual weights, proximate, and mineral composition of both types of *S. domestica* fruits are shown in Table 1.

The fruits are apple- and pear-shaped pomes. Despite being edible, when green, the fruits are very rough to the taste and should be eaten overripe to lose their astringency. Morphologically, both types measure 5 to 6 cm in diameter in the immature state, decreasing to 3 to 4 cm in the mature state. The color of the exocarp, initially yellowish with a slight or pronounced red blush on the sun-exposed side, changes to very dark brown at maturity. The skin is mostly thin and smooth; in the ripe fruits, it retains the texture but is easy to eat. The pulp in unripe fruits is whitish and firm, with a single hard bone, and it is acidic and astringent. The pulp of overripe *S. domestica* fruits has a brownish color, and it is soft in texture and fragrant and sugary in flavor.

Fruits of the apple-shaped type have a statistically higher caliber (*p* < 0.05) than fruits of the pear-shaped type. On average, the unit weight of apple-shaped *S. domestica* fruits is 41.9% higher than that of pear-shaped fruits.

The moisture content was the most stable fruit parameter (less variable). The water content in both *S. domestica* types was similar, around 65.5%, and the dry matter average was the percentage difference (34.5%). According to the statistical analysis, there were no significant differences (*p* > 0.05) between the moisture and dry matter contents of the two types. The ash or total mineral content in the edible fraction of *S. domestica* fruits was very similar between the two types. There were no significant differences (*p* = 0.0897) in this parameter between the apple-shaped and the pear-shaped types; the concentrations of total minerals were 0.51% and 0.60%, respectively. Despite the soft texture of the fruits, both *S. domestica* types had a high total fiber content, ranging from 2.52% (apple-shaped) to 4.91% (pear-shaped). The differences were not statistically significant due to the wide discrepancy in coefficient of variability (CV) observed in this parameter for the apple type (30.79%). Despite the high coefficients of variability, the fat contents showed statistically significant differences (*p* = 0.0244), with higher concentrations for the apple type (0.67%) compared to 0.43% for the pear type of *S. domestica* fruits. The protein content was slightly higher in the *S. domestica* pear-shaped fruits (0.68%) than in the apple-shaped fruits (0.54%), but the differences were not statistically significant between the protein values of the two types, possibly due to the low concentrations and high variability of this parameter in the fruits. Carbohydrates are the main nutrients in *S. domestica* fruits. Their level was higher in the pear type at 30.17% versus 27.91% for the apple type, although these values did not show statistically significant differences. This nutritional composition resulted in an energy value of 129.65 and 132.20 kcal 100 g^−1^ fw for *S. domestica* fruits of the apple and pear types, respectively, although the differences found were not statistically significant (*p* = 0.3933). These values show that these are moderately caloric fruits.

The content of inorganic components or mineral elements is a characteristic that has a very positive influence on the nutritional value of the fruit [34]. Of the thirteen mineral elements analyzed, the concentrations of the heavy metals Pb and Hg and the elements Cr and Se were insignificant (<0.01 mg 100 g^−1^ fw) for both types of *S. domestica* fruits. In both fruit types, the most abundant mineral was potassium. Fruits of the pear-shaped type contained 16.11% more of this macro-element, with the differences found being statistically significant (*p* = 0.0049). The second most abundant mineral element in *S. domestica* fruits was calcium, with 44.59 mg·100 g^−1^ fw (apple) and 43.93 mg·100 g^−1^ fw (pear). The calcium concentrations were very similar in the two types, with no significant differences between the values (*p* = 0.7894). A similar trend was observed with the third mineral element (phosphorus) present in the fruits of *S. domestica*. The concentrations were higher for the apple-shaped type (18.16 mg·100 g^−1^ fw), with no statistically significant differences observed (*p* = 0.8006). Sodium was the macro-element mineral with the lowest content in *S. domestica* fruit, with contents close to 7 mg per 100 g of fresh fruits, with no significant differences between types (*p* = 0.3100). On the other hand, significant differences were observed between the magnesium contents of apple- and pear-shaped types (*p* = 0.0308), where pear-shaped *S. domestica* fruits contained, on average, 11.8% more Mg. In the micro-elements (Mn, Fe, Cu, Zn) of *S. domestica* fruits, the highest concentrations were found for iron. The iron values ranged between 0.47 mg 100 g^−1^ fw (pear-shaped type) and 1.42 mg 100 g^−1^ fw (apple-shaped type), with the differences being statistically significant (*p*-value = 0.0029). The concentrations of zinc contents in *S. domestica* fruits were also significantly higher (*p*-value = 0.0069) for apple-type fruits (0.109 mg 100 g^−1^ fw) compared to 0.061 mg 100 g^−1^ fw for pear-shaped fruits. For copper, the trend was similar to those observed for iron and zinc, i.e., higher concentrations (0.081 mg 100 g^−1^ fw) in the apple-shaped type of *S. domestica* fruits compared to 0.063 mg 100 g^−1^ fw in the pear type, with the differences found being statistically significant (*p* = 0.0373). Finally, the manganese concentrations did not show significant differences (*p* = 0.5740) between the values of both *S. domestica* types, possibly due to the high variability found in the values of the apple-shaped type.

### 2.2. pH, Soluble Solids Content, Total Acidity, Total Sugars, and Glucose Composition of S. domestica Fruits

The results of the average values for the pH, soluble solids content, total acidity, total sugars, and glucose composition of both types of *S. domestica* fruits are shown in Table 2. 

The parameters of pH, soluble solids content, total titratable acidity, total sugars, and glucose analyzed in *S. domestica* fruits are related to the flavor attributes of the fruits [35]. The acidity of the ripe fruits was determined by both pH value and total titratable acidity. In both cases, statistically significant differences were observed. The pH values of the fruits of the apple-shaped type were significantly lower (pH = 3.56) compared to those of the pear-shaped type (pH = 3.98). The fruits of the pear type were the least acidic, with a total acidity of 0.786 g malic acid·100 g^−1^ compared to 1.173 g malic acid·100 g^−1^ for the fruits of the apple type, which were significantly more acidic. There is a logical inverse relationship between pH values and total acidity. The most acidic fruits have a higher total titratable acidity value and a lower pH value.

In general, the acidity of *S. domestica* fruits is low, which leads to a high total sugars/total acids ratio and sweet sensations in the fruit. The apple-shaped type of *S. domestica* fruits stands out for their higher concentration of total sugars (14.19 g 100 g^−1^ fw), with significant statistical differences (*p*-value = 0.0029) compared to the pear-shaped type (11.42 g 100 g^−1^ fw). This trend is also evident in the case of individual sugar (absolute glucose values). The glucose concentrations of apple type *S. domestica* fruits contained almost 1 g more of this sugar per 100 g of edible fresh fruit fraction. That is, the glucose contents of apple-type *S. domestica* fruits were significantly different (*p* = 0.0044), containing 15.73% more of this sugar. Sugars in the fruits represented approximately 70% of the soluble solids content. The total soluble solids content (SSC) in *S. domestica* fruits was very high (16.57 °Brix and 16.29 °Brix for apple and pear types, respectively), with the differences between both being non-significant (*p* = 0.7994).

### 2.3. Total Phenolic Content and Antioxidant Activity by DPPH Assay of S. domestica Fruits

Results of total phenolic content and antioxidant activity by DPPH assay of *S. domestica* fruits, in both types are shown in Table 3, expressed in caffeic acid equivalents (ECA). Polyphenols were by far the most abundant dietary antioxidants due to their presence in the fruits. These parameters are a good representation of the fraction of bioactive components in the fruits [36].

The total antioxidant content, expressed as µmol equivalent Trolox (TE) in the fresh *S. domestica* fruits, was higher in the apple-shaped type, with 283.67 μmol ET·g^−1^ compared to 170.58 μmol ET·g^−1^ in the pear-shaped fruits. However, these differences were not statistically significant at the 95% confidence level. The total polyphenolics content ranged from 93.67 (apple-shaped type) to 107.40 (pear-shaped type) (mg ECA·100 g^−1^). The difference of 12.78% found between both types was not statistically significant (*p* = 0.3265).

The total phenolics were higher in the apple-shaped type compared to the pear-shaped type, whereas the antioxidant activity measured by the DPPH assay was the opposite. Phenolic compounds, in general, may increase the results of the DPPH assay because they can scavenge free radicals as well as superoxide and hydroxyl radicals. However, phenolic compounds are not the only factor influencing antioxidant value. Other bioactive substances, such as vitamin C or carotenoids, which were not evaluated in the present study, may also contribute to the antioxidant activity. In addition, synergistic effects may also occur between some components, which favor the DPPH assay.

### 2.4. Volatiles Profile of S. domestica Fruits

The volatile composition of *S. domestica* has been determined in three stages of ripening to assess its organoleptic characteristics related to aromas and flavors. No references have been found on the aromatic profile of *S. domestica* fruits; therefore, in the absence of references for this type of study, the aromatic profile of a fresh, ripe apple and the aromatic profile of quince jam were used as a control since they are foods that allow the results obtained to be compared.

A total of 51 volatile aromatic compounds were detected. Table 4 details the qualitative analysis of aroma compounds (mean value ± standard deviation) of the GC-peak area of ester, sesquiterpene, sulfur derivative, monoterpene, and alcohol volatile components in *S. domestica* fruits from the three ripening stages (unripe or green, intermediate, and ripe) for each type (pear- and apple-shaped). Table 5 shows the same values for the rest of the volatile components (aldehydes, ketones and methyl ketones, carotenoids, aliphatic and aromatic hydrocarbons, alkanes, carboxylic acids, heterocycles, and other compounds). The peak area of the aromatic components for the two controls (apple fruit and quince jam) is also shown.

In the case of the *S. domestica* apple-shaped type, 42 compounds were detected in the unripe stage (ImF), 45 compounds in the intermediate stage (IF), and 32 compounds in the ripe stage (RF). In the case of the pear-shaped type, a total of 43, 44, and 30 compounds were detected for each of the ripening stages, respectively. These results show that the intermediate stage of ripening is the most complex due to the abundance of aromatic compounds, while the fruits in the ripe stage present a lower number of volatile compounds, regardless of the type. In contrast, in the analysis of a Red Delicious apple and quince jam, only 10 and 14 aromatic compounds were detected, respectively. This decrease in the case of the control foods is due to the commercial nature of apple fruits, which have undergone processes with temperature changes in which they may have lost part of their original volatile fraction and, in the case of quince jam, because it is a processed food, and the effect of the processing temperature may have influenced the loss of the volatile components of the original fruit.

The different aromatic compounds were grouped into 13 chemical families. Table 6 represents the mean value percentage GC-peak area for each of the chemical families present at each moment, compared to the total peaks at that moment, in *S. domestica* fruits for the two types and three states of maturity and in the control foods. It was observed that alcohols are the main aromatic chemical compounds in *S. domestica* fruits, with the alcohol fraction being higher in the case of apple-shaped fruits and in the ripe state for both types. For apple-shaped *S. domestica* fruits, alcohols represented 57.52% of the total aromatic fraction in the ImF state, 62.57% in the IF state, and 74.25% in the RF state, and for pear-shaped fruits, alcohols represented 38.24% of the total aromatic fraction in the ImF state, 40.18% of the aromatic fraction in the IF state, and 46.75% in the RF state. Alcohols were the second-highest component in the control foods. Fresh Red Delicious apples concentrate their aromatic components in esters, while quince jam concentrates them in the aldehyde chemical family. Aldehydes are also a major chemical component that appears in the second-highest concentration in the fruits of *S. domestica*. For apple-shaped fruits, aldehydes represented 16.81% of the total aromatic fraction in the ImF state, 19.26% in the IF state, and 6.12% in the RF state, and for pear-shaped fruits, aldehydes represented 26.36% of the total aromatic fraction in the ImF state, 26.60% in the IF state, and 21.92% in the RF state. In both cases, the values followed a bell-shaped temporal evolution, with a maximum percentage in IF. Aldehydes also appeared in the control foods. The third group of chemical compounds was heterogeneous (others) and mainly owed its percentage strength to 2,4,5-trimethyl-1,3-dioxolane, which belongs to the acetal compounds. These compounds could be formed through the interaction between aldehydes and alcohols [37], and their aroma is associated with green and phenolic notes. 

The rest of the aromatic chemical families were found in percentage concentrations below 5%, with sesquiterpenes standing out in pear-shaped fruits in the unripe state. Sesquiterpenes showed an identical aromatic profile in both *S. domestica* fruit types, with higher concentrations in ImF that decreased as the ripening stages progressed. In the case of Red Delicious apples and quince jam, the total sesquiterpene levels were very low compared to those present in *S. domestica* fruits. Esters were present in all ripening stages of *S. domestica* fruits and in both types, with higher concentrations in the RF state; it was observed that they are compounds that are synthesized during the ripening process. These chemical compounds were not detected in quince jam and in the case of the fresh apple. The values of this chemical family were strongly influenced by the presence of hexyl acetate. Sulfur-derived compounds were at their maximum point during ImF in pear-shaped fruits, and with the ripening process, they degraded or volatilized, leaving a marked absence in RF. For apple-shaped fruits, the highest percentage value was found in IF, and it also disappeared in RF. 

Monoterpenes did not appear in the control food. For *S. domestica* fruits, the highest concentrations were also found in the pear-shaped type. Specifically, in this type, concentrations appeared in all three stages of ripening, being notably higher in IF and decreasing in RF, while in the apple-shaped type, the monoterpene components disappeared completely in RF. Ketone components were not detected in pear-shaped fruits at the ImF ripening stage or apple-shaped fruits at the IF ripening stage. The punctual appearance of these components in low concentrations was possibly due to the oxidation of some alcohols. They were not present in the Red Delicious apples, nor did they appear in quince jam. In both types of *S. domestica* fruits, the highest contents were found in the RF state. Carotenoid compounds showed concentrations close to 1% in the pear-shaped type and 0.5% in the apple-shaped type. These components only appeared in the ImF and IF stages, disappearing in both types of *S. domestica* fruits at the RF stage, possibly due to the degradation of these pigments. These compounds did not appear in the fresh apple fruit and were significantly high in quince jam.

Aliphatic and aromatic hydrocarbons were not detected in the control foods. In *S. domestica* fruits, they appeared as the fruit ripened, except for pear-shaped fruits. In apple-shaped fruits, these compounds were more present in the ImF state, decreased in IF, and increased in RF. The alkanes that did not appear in the control foods were present in the ImF of *S. domestica* fruits, disappearing in the later stages for pear-shaped fruits and gradually reducing in apple-shaped fruits but being present in RF. Carboxylic acids are compounds formed by the oxidation of alcohols and other oxygenated compounds. They present an evolutionary synthesis during the ripening of *S. domestica* fruits, with an increase in percentage values observed in RF. Red Delicious apples presented low levels of these fatty acids, while for quince jam, the concentrations were high. These variations were mainly due to the presence of lauric acid. The heterocyclic chemical family of compounds was not present in the control foods. The evolution of their concentrations was similar for both types of *S. domestica* fruits, with an increase in concentrations as the ripening process occurred.

Finally, at the RF stage, pear-shaped fruits did not show the presence of alkanes, aliphatic and aromatic hydrocarbons, sulfur-derived compounds, and components of the carotenoid family. Sulfur-derived compounds, monoterpenes, and carotenoids were not detected in apple-shaped fruits in the RF stage.

The individual compound with the highest presence in most *S. domestica* fruits is 1-hexanol; this component represented 25.38% of the total aromatic fraction in the ImF state, 21.88% of the aromatic fraction in the IF state, and 18.94% in the RF state of pear-shaped fruits. For apple-shaped fruits, the presence of 1-hexanol was higher, being 35.31% in ImF, 28.13% in IF, and 26.20% in RF. 1-hexanol was also detected in the two control foods. The acetal compound 2,4,5-trimethyl-1,3-dioxolane represented 13.84% of the total aromatic fraction in the ImF state, 25.50% in the IF state, and 10.75% in the RF state of apple-shaped fruits; and for pear-shaped *S. domestica* fruits, it represented 22.41% of the total aromatic fraction in ImF, 24.04% in IF, and 20.13% in RF. The third individual major component was phenylacetaldehyde, which is an aromatic aldehyde related to the sweet and caramelly attributes; this component represented 8.67% of the total aromatic fraction in the ImF state, 8.38% in the IF state, and 1.11% in the RF state of apple-shaped fruits. For pear-shaped fruits, the presence of phenylacetaldehyde was higher, being 8.65% in ImF, 12.61% in IF, and 8.55% in RF. 2,4,5-trimethyl-1,3-dioxolane and phenylacetaldehyde are aromatic components exclusive to the fruits of *S. domestica* in both types since they were not found as aromas in Red Delicious apples or quince jam.

The individual components that increased in concentration in ripened *S. domestica* fruits compared to the total were butyl acetate, methyl nonanoate, benzaldehyde, nonanal, γ-nonalactone, and 2-pentylfuran only for pear-shaped fruits and hexyl acetate, ethyl octanoate, 3-methyl-1-butanol, 2-methyl-1-butanol, 1-heptanol, benzyl alcohol, phenylethyl alcohol, 1,3-octanediol, 2-heptanone, β-methylnaphthalene, lauric acid, γ-decalactone, and camphor in both types. Some of these compounds are synthesized during the ripening process.

The aromatic complexity was higher in *S. domestica* fruits. For the control food, fresh commercial apples showed an important reduction in the number of aromatic components, and the concentration of hexyl acetate was notable. Curiously, the fruits of *S. domestica*, regardless of the shape and ripening stage, were not characterized by the presence of this component. In the case of quince jam, the concentrations of phenethyl alcohol and 2-heptenal, which have been described as having a green fruit smell and a sweet, fresh, or apple-like taste, stood out. Phenethyl alcohol appeared in low concentrations in the fruits of *S. domestica*, and *cis*-2-heptanal only appeared in the initial stages of ripening and disappeared in RF. It seems that they play a minor role in the aroma of the fruits of *S. domestica*.

To try to establish relationships between the different chemical families of the volatile compounds studied and to evaluate their proximity to the components of fresh apple and quince jam, a factor analysis was carried out. A model with four main factors was obtained, which explained 96.57% of the variability in the original data.

**Figure 2:**
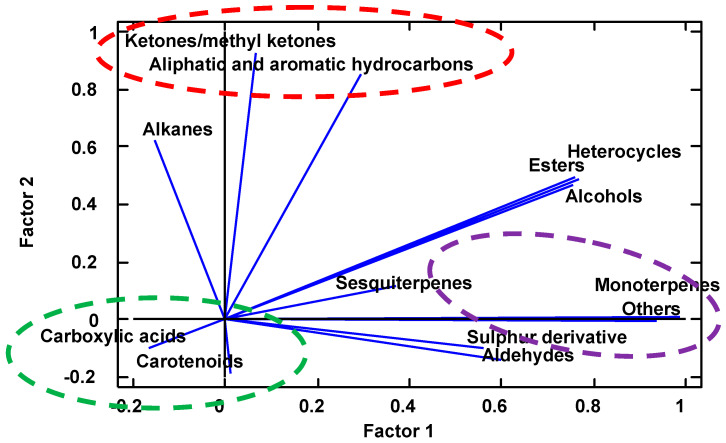
Scatter plot of the weights of the main factors of the aroma chemical families in *Sorbus domestica*.

The samples studied in this work allow the classification of three categories (Figure 2): one with low values of factor 1 and low values of factor 2 (green line), one with high values of factor 1 and low values of factor 2 (purple line), and one with high values of factor 2 and neutral values of factor 1 (red line).

**Figure 3:**
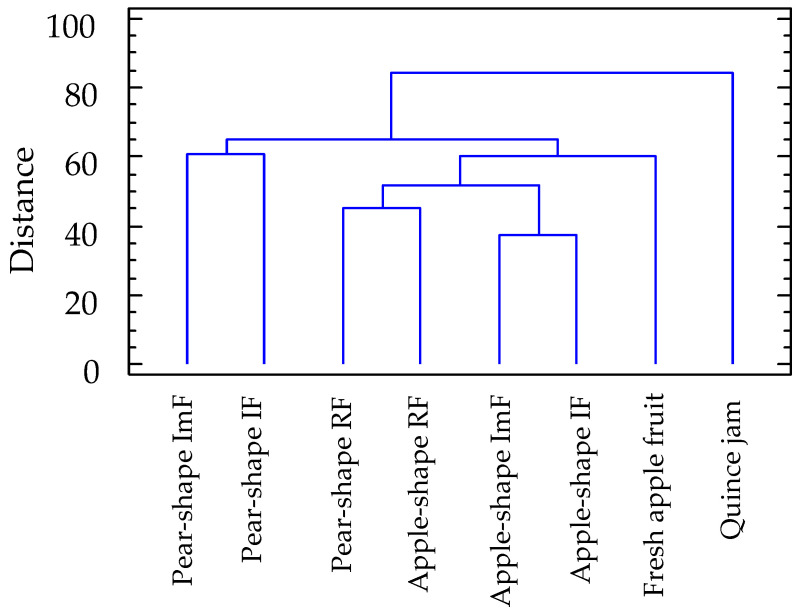
Cluster identification using the volatile profile.

The cluster analysis carried out on all the volatile components detected in the study expanded the classification. The analysis created one cluster (Figure 3) where the quince jam samples stand out from the rest. In addition, the apple-shaped *S. domestica* samples bridged the gap with the aromatic components found in fresh apples and with the ripeness states of both types of *S. domestica* fruit. The samples with the greatest distance were those of *S. domestica* fruits in the unripe states.

## 3. Discussion

The scientific literature provides valuable information about the species *S. domestica*. Much of the research focuses on the distribution of its trees in the territory [38,39,40,41,42,43,44], including its distribution in the Iberian Peninsula [22,45]. Due to its genetic diversity and its adaptation to heat and drought, under conditions of climate emergency, the *S. domestica* species can be an alternative for the regeneration of forest and urban spaces [46,47,48]. There are studies that describe the contribution of *S. domestica* fruits and leaf extracts in folk remedies due to their astringent, anti-inflammatory, antiatherogenic, antidiarrheal, antidiabetic, diuretic vasoprotective, and vasorelaxant activities [24,33,49,50]. Although, in recent years, their nutritional and biochemical composition has been studied, to the best of the authors’ knowledge, this information has not been found for fruits from the Iberian Peninsula, including the volatile composition and distinguishing between fruit polymorphisms.

Špíšek et al. [40] classified six categories of fruits according to their shape, including the pyriform and conical ones in the present study. Other authors [51] have identified the same two shapes found in the northeast of the Iberian Peninsula in the Serbian area, with a greater predominance of apple-shaped fruits. During the overripening process, the size decreases, but the shape of the fruits is maintained.

The unit weight of both types of *S. domestica* fruits would be within the category of small fruits according to the criteria of Miletić and Paunović [51]. According to the results of Tas et al. [52] on the typing of 10 *S. domestica* genotypes in an area of Turkey, there is high variability in the unit weight of the fruits, ranging from 5.4 g to 12.5 g. The caliber of fruits from other studies [53,54] corroborate that the weights of the fruits in this study are of small caliber, although within the ranges of variability. The size of the fruit could be influenced by climatic and edaphic factors. In extreme drought conditions, during the stage of intensive growth and fruit ripening (months of June, July, August), the unit weight of the fruits and their size can be reduced.

The quantification of moisture content is one of the most widely used techniques in food control, processing, and preservation. Water is the major component present in foods and also the major component in both types of *S. domestica* fruits. *S. domestica*’s moisture content exceeded that of other *Sorbus* species as well as the Bulgarian *S. aria* with 54.50% [55]. Altuntaş et al. [56] investigated the moisture content during the different ripening periods of *S. domestica*, finding that when the fruits reach the overripe state, the moisture content is 71.84%, a value higher than that found in the present study. The characterization study carried out by Poljak et al. [57] provided moisture content values in *S. domestica* fruits ranging from 56.8% to 69.50% and showed that in general, Mediterranean populations had somewhat lower water content values than continental populations.

If the water content is compared with conventional fruits such as apples, pears, quinces, or plums, where the moisture contents range between 82 and 86%, the moisture content of *S. domestica* fruits is low [58]. The dry matter, by contrast, is relatively high, and it is a positive attribute in jam and jelly production, so the fruit performance here could be very productive.

Ash content provides information about the total mineral concentration in the fruits. The ash content found in both *S. domestica* fruit types shows a low value when compared to the values in *S. aria* from Bulgaria with 2.53% [55] and the values, expressed in dry weight, in *S. domestica* from Croatian continental and Mediterranean regions [57], slightly lower (0.98%) than those found in a collection of fruits from Bulgaria [25] and similar to the ash contents of conventional pear and apple fruits [58].

The fiber values for pear-shaped *S. domestica* fruits are close to those of whole and fresh plums, apples, or pears. The fiber content of apple-shaped fruit is close to the values found for fresh quinces, which are characterized by a high fiber content and a high level of pectin [57]. Ognyanov et al. [25] indicated that pectin (soluble fiber) can form 48% of the polysaccharides of *S. domestica* fruits and that starch, whose content significantly decreases in the ripened fruits, constituted a minor part of the total carbohydrate content. The intake of 100 g of fresh pear-shaped fruit will provide about 10.08% of the recommended amounts of dietary fiber, a value that rises to 19.64% when it comes to apple-shaped fruits.

*S. domestica* fruits maintain the general trend of being low-fat foods. The crude fat of apple-shaped *S. domestica* fruits is similar to that indicated in the literature [25,57], and pear-shaped *S. domestica* fruits have a significantly lower content of this nutrient. The protein levels in fresh fruits also follow a trend of low concentrations. The values found in this study are within the range indicated by other authors [57] for the fruits of *S. domestica* and according to the databases [58], with a range of protein values similar to those in fruits such as apples, quinces, and pears.

Carbohydrates constitute a major part of dry matter constituents in both *S. domestica* fruit types. The values found in this research are slightly lower than the values, expressed in dry matter, found in the literature [25]. The intake of 100 g of apple-shaped *S. domestica* will provide about 21.47% of the daily requirements of total carbohydrates, and the intake of 100 g of pear-shaped fruits will provide 23.18%. Carbohydrates have a protein-sparing effect and are central to the postharvest respiratory metabolism in many fruits, being the most important material base for obtaining good fruit storage characteristics [59] and a highly interesting attribute for the use of *S. domestica* fruits to produce jams.

The energy value of *S. domestica* fruits is high compared to common fresh fruits such as apples and pears [58], possibly due to the higher amount of dry matter and high carbohydrate content. These nutrients provide 86.11% of the total energy of apple-shaped *S. domestica* fruits and 91.16% of pear-shaped ones. In any case, the caloric value of 100 g of fruits, regardless of the shape, provides 6.5% of the average daily caloric requirement of 2000 kcal. These results coincide with those cited by other authors [25], so they can be used in healthy non-caloric diets.

Minerals are important for all living organisms. An imbalance of macro and microelements in the organism causes metabolic disorders and health problems. According to Steinnes [60], some of the most serious health problems are related to an inadequate supply of trace elements. The mineral content in fruits can vary according to the edaphoclimatic conditions and the state of maturity of fruits at harvest [61]. Potassium (185.56 and 221.19 mg 100 g^−1^ fw for apple-shaped and pear-shaped, respectively) and calcium (approximately 40 mg 100 g^−1^ fw in both types) were the most abundant macroelements. This distribution coincides with what was reported by Majić et al. [27] in fruits collected in Croatia, and the concentration is higher than that shown by other authors [25]. The rest of the macroelements (P, Mg, Na) are found in the fruits in smaller quantities and influence the cross-relationships of these elements to maintain the electrolyte balance. Common fresh pear and apple fruits contain lower macro- and microelements than *S. domestica* fruits [58]. The microelements (Cr, Cu, Zn, and Fe) have also been detected in other studies with *S. domestica* [25,27], with Fe being the most concentrated in the fruits. These mineral elements are essential components, and some are part of the enzymatic systems, as evidenced by the fact that their deficiencies have profound effects on metabolism and tissue structure [62]. The iron content in the *S. domestica* fruits was distinctly superior in comparison to that of common fruits such as apples, apricots, and plums [58].

The sugar and acid parameters will determine the organoleptic characteristics of *S. domestica* fruits. The difference in this parameter may be caused by diverse factors as genotype, geographical location, ecological factors, and soil characteristics. The pH ranges of the two types were found to be lower than 4. Other authors have found higher values [25,52,54,56], but pH values of 3.2 were also reported [63], so the fruit juice pH obtained in this study is within the existing literature. The literature also offers a wide range of variability regarding SSC (from 11.17 to 20.20 °Brix) [51,52,56], which includes the fruits of the present study. In some regions, fruits with values higher than 30 °Brix have been found [29,64]. The titratable total acidity of *S. domestica* fruits also shows a wide variability, from 0.42 (g malic acid 100 g^−1^ fw) [53] to higher values [29,63]. It is observed that the acidity values determined in this study are among the results reported in the literature. In general, the increase in pH and decrease in total acidity are related to the ripening process of the fruits, as there are lower concentrations of organic acids, as observed in the specific case of the apple [65]. However, in the specific case of the *S. domestica* fruits in this work, the differences found are likely due to morphological differences since both fruits were collected at the same time in the same area and were subjected to the same overripening process. The total sugars represented a large part of the total carbohydrate content of *S. domestica* fruits. The main sugar found in both ecotypes was glucose. This trend does not match with that described by other authors [25,55] from Bulgarian fruits of *S. domestica*. The main sugar found in these studies was fructose (3.63 and 5.7 g·100 g^−1^ respectively), followed by glucose (2.73 and 4.0 g·100 g^−1^ respectively). This increase in glucose content could be explained by the overripening process influenced by the enzymatic action that degrades carbohydrates [66]. The absence or low contents of sucrose in *S. domestica* fruits makes them suitable for use in dietary nutrition.

*S. domestica* fruits are highly astringent when overripe, so they are edible only when they are overripe [52]. Climacteric fruits, such as *S. domestica*, experience an increase in ethylene production when they are ripening. This triggers the synthesis of enzymes involved in the process of pulp decomposition, cell wall softening, and carbohydrate metabolism [67]. Starches are broken down into simpler sugars. At the same time, enzymes such as sucrose synthase and malic enzyme facilitate the accumulation of sugars such as glucose and fructose and a decrease in acidity due to the degradation of malic acid. The synthesis of pigments, such as carotenoids and anthocyanins, also takes place [68]. These processes result in brown-colored fruits that are soft in texture, with greater sweetness and high aromatic content.

The health benefits of *S. domestica* fruits have been linked to their polyphenolic content and antioxidant value [24,69]. The beneficial effects of the bark, seeds, and leaves of *S. domestica* trees on human health have also been established [24,27,70,71,72], and the evolution of polyphenolic content during fruit ripening has been analyzed [30]. The variability of the results is very high. The total polyphenol concentrations in this research are higher than those found by some authors [52] and lower than those found by others when the results are expressed in equivalents other than caffeic acid [25,29,57].

*S. domestica* fruits have been shown to have a high polyphenolic concentration, and these compounds [73] may be responsible for the antioxidant assay of the fruits [74]. Antioxidant activity is the ability of a compound to eliminate reactive free radicals generated by oxidative stress. There is high variation in the results of the literature. When comparing our results with those reported by other authors [25,27,57], it can be observed that there are variations, which may be due to differences in result expressions, extraction methods, and solvents, which cause different yields of total phenols and, therefore, a variation in antioxidant activity [24]. The phenolic and antioxidant profiles depend largely on the specific *S. domestica* fruit species [75] and the state of ripeness. Termentzi et al. [30] demonstrated that the fresh yellow (unripe) fruits of *S. domestica* have the highest antioxidant activity, while fully ripe brown fruits, which are ready to eat, show lower antioxidant activity, possibly because overripe fruits have lost astringency related to the presence of tannins, substances that contribute positively to antioxidant activity.

Since there is no literature on the volatile composition of *S. domestica* fruits, this research is pioneering in this regard. However, some authors [23,76] have tried to establish a volatile profile in distilled beverages produced from *S. domestica*, identifying the presence of almost 100 aromatic compounds. The presence of aromatic components in *S. domestica* fruits has two fundamental origins: on the one hand, the production of volatile compounds beginning from fruit-set, which is modulated by the genetic and environmental load and originates the volatile fraction at the immature stage (ImF), and on the other hand, the degradation and synthesis processes that occur in the components and give rise to the final aromatic composition of the fruits. For example, sulfur derivatives that appear at the intermediate stage of ripening possibly come from the transformation of sulfurous amino acids in the fruit [77], compounds that due to their high volatility may disappear at the optimum stage of ripening.

Alcohols are generally components produced after the anaerobic fermentation processes of fruit sugars. They usually have intense and pungent odors that contribute significantly to various flavor compounds [78]. In addition, alcohols are precursors in the synthesis of aldehydes and ketones, fatty acids, and, consequently, esters. The present study has shown that *S. domestica* fruits contain abundant levels of alcoholic flavor compounds, coinciding with what has been shown for apples and ciders by other authors [79]. Alcohols are the most important chemical components in the fruits of *S. domestica*, mainly due to the presence of two alcoholic components (2-methyl-1-butanol and 1-hexanol) that are found in both types of fruits and in the three stages of ripening. 2-methyl-1-butanol confers a fruity flavor and sweetness, like whisky and cocoa [80], while 1-hexanol gives a fruity and floral-like flavor and a sweet smell to the fruits. 1-hexanol is a volatile alcohol with a green leaf flavor and is a major component of essential oils extracted from different tropical fruits [81]. Phenethyl alcohol is another important component in the IF and RF ripening states. It is a product of the degradation of phenylpropanoids and is characterized by a floral aroma reminiscent of roses [82].

2,4,5-Trimethyl-1,3-dioxolane has been identified as a secondary metabolite, so it may play a role in cellular defense. It is a compound that has been identified in different foods that undergo fermentation processes, such as in the production of wine, beer, and vinegar. It has also been detected in berries and is related to phenolic and astringent/drying flavor notes [83].

The 6-methyl-5-hepten-2-one component belonging to the carotenoid family is not found in Red Delicious apples, and it disappears in the RF state for both types of *S. domestica* fruit, whereas in the pear-shaped fruit, it has a relative presence. And in quince jam, its concentration is significantly high, possibly because cooking for the preparation of quince jam can increase the carotenoid content due to better extraction via the exposure of the cellular content [84]. 1,3-octanediol has been another prominent individual component of the volatile fraction. This alcohol has been widely identified in apples and ciders [85,86,87], but in this research, it has only been detected in the three stages of ripening of the fruits of *S. domestica*.

In general, it is observed that the aroma profiles of immature *S. domestica* fruits were dominated by aldehydes, while in the overripe stage, the fruit accumulated abundant esters, alcohols, and sesquiterpenoids. Aromatic components that may be related to foreign and/or unpleasant aromas, such as diallyl disulfide, α-methylnaphthalene, tetradecane, and hexanoic acid, appear only in the immature stages of *S. domestica* fruits and may be precursors of other aromatic components.

The aromatic complexity is higher in *S. domestica* fruits. Fresh commercial apples (control food) have a smaller number of aromatic components, and the concentration of hexyl acetate is notable. This substance contributes to the perception of the sweet and fruity aroma of apples [88]. Curiously, the fruits of *S. domestica*, regardless of the shape and time of ripeness, are not characterized by the presence of this component. In the case of quince jam, the concentrations of phenethyl alcohol and 2-heptenal, which have been described as having a green fruit smell and a sweet, fresh, or apple-like taste, stand out. Phenethyl alcohol appears in low concentrations in the fruits of *S. domestica*, and 2-heptanal appears only in the initial stages of ripening and disappears in RF; therefore, it consequently contributes at a low level to the aroma of the fruits of *S. domestica*.

Factor analysis can help to establish the relationships between aromatic components (Figure 2). The group with low values of factor 1 and factor 2 (green line) is mainly characterized by its high content of carotenoid compounds and fatty acids; this group would include quince jam. The group with high values of factor 1 and low values of factor 2 (purple line) is mainly characterized by high concentrations of monoterpenes, sulfur-derived components, the major component 2,4,5-trimethyl-1,3-dioxolane (included in the group of others), and even by the strength of sesquiterpenes; this group includes pear-shaped *S. domestica* fruits. The third category, with high values of factor 2 and neutral values of factor 1 (red line), is represented by high values of aliphatic and aromatic hydrocarbons and ketones; this group includes apple-shaped *S. domestica* fruits.

The rest of the aromatic chemical groups do not allow for establishing discriminant relationships since they are very common components of the aromatic fraction. The results show that the fruits of *S. domestica* have a small fraction related to apple fruits or quince jam, which opens new research opportunities to establish similarities with other fruits, possibly with tropical fruits.

## 4. Materials and Methods

### 4.1. Vegetal Material and Sample Preparation

*S. domestica* fruits were collected in an immature state, at random, from all parts of the tree canopy under wild conditions in healthy trees located in the northeast of Iberian Peninsula (Valladolid, Spain). The collection of immature fruits (green and hard) was carried out at the end of September 2019.

**Figure 4:**
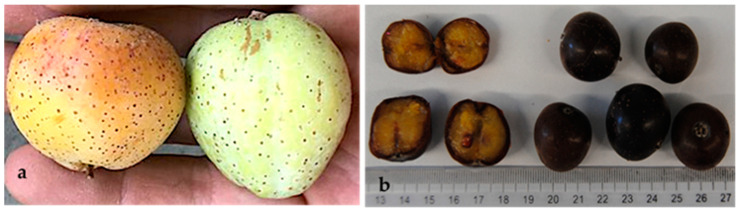
*Sorbus domestica* fruits apple-shaped type: (**a**) complete unripe fruit; (**b**) cross-section of ripe fruit.

**Figure 5:**
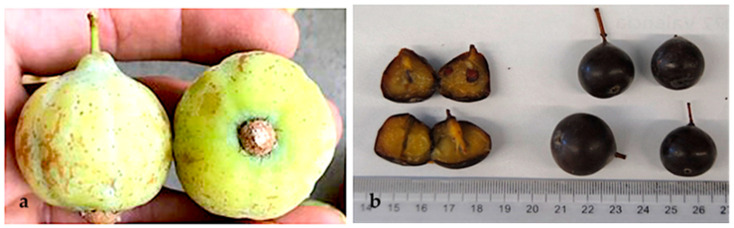
*Sorbus domestica* fruits pear-shaped type: (**a**) complete unripe fruit; (**b**) cross-section of ripe fruit.

This study includes ripe fruits *S. domestica* (apple- and pear-shaped fruits) that were identified and sorted by their morphology at the time of fruit collection and denominated the apple-shaped type (Figure 4) and the pear-shaped type (Figure 5). Approximately 2 kg of fruits of each type was collected for analysis.

Weights of individual fruits (W) were obtained by weighing them on an analytical balance CB-Junior-Cobos (Valencia, Spain) with an accuracy of ±0.001 g.

The fruits were washed and placed on a bed of straw to ripen. The determinations of the volatile profile were carried out at three moments: 1. Immature fruits (ImF), three days after harvest. At this stage, the skin showed green shades, and the pulp was green-yellow. 2. Intermediate fruits (IF), fifteen days after harvest. The fruits were at an intermediate stage of ripening (brown skin color and firm pulp, but with color turning) 3. Ripe fruits (RF), thirty days after harvest. The fruits were overripe and fermented (with brown skin and pulp and very creamy pulp). The maturity was determined by full orange-red coloration. The determinations of the proximal analysis were carried out on RF because it is the state of consumption of these fruits. At the time of analysis, the fruits were cut longitudinally, and the seeds were removed. For analytical quantifications, the entire edible fraction (skin and pulp) was used.

Each fresh sample was divided into three fractions. One was used for drying the fruits, which were subsequently crushed until a homogeneous mixture (250 µm) was obtained. The dried and crushed fraction of the fruits was used to determine the proximal and mineral composition. A second fraction of the fresh fruit samples was squeezed using a domestic juice extractor. Fresh juice was used to determine total titratable acidity, pH, total soluble solids, and total sugar content and glucose. Another part of the fresh fruit was used to obtain methanolic and aqueous extracts to determine the total antioxidant activity the total polyphenol content, respectively. To determine the volatile profile, the fruits from each phase of ripening were used. And to compare the results, fresh Red Delicious apple fruits and quince jelly were used. Three replicates were performed for each analysis.

### 4.2. Proximal and Mineral Composition

Proximate composition from *S. domestica* fruits was carried out by official methods [89]: moisture (M) and dry matter (AOAC 984.25) using a stove J.P. Selectam (Barcelona, Spain) at 70 ± 0.1 °C until constant weight; proteins (AOAC 984.13) by the Kjeldahl method using the Foss Tecator 2100 Kjeltec distillation unit (Hillerød, Denmark); fat (AOAC 983.23) using a Foss Soxhlet model ST243 Soxtec^TM^ from Labtec^TM^ line (Hillerød, Denmark); fiber (AOAC 991.43) quantifying the residue that persists after two successive hydrolyses, one acidic and the other alkaline; and ashes (AOAC 923.03) by calcination in a Carbolite CWF 1100 chamber furnace at 550 °C. The carbohydrate content was calculated by difference. The total energy was calculated by multiplying by 9 kcal the grams of fat, 4 kcal the grams of protein and carbohydrates and 2 kcal the grams of fiber by each 100 g of fresh *S. domestica* fruits. The results are expressed as g·100 g^−1^ of fresh weight (fw).

The individual mineral composition was determined by inductively coupled plasma emission spectroscopy (ICP-EOS), using the ashes dissolved and settled with concentrated HCl until a 2% HCl solution. The equipment used was Agilent ICP-EOS 710 (700 series ICP-OES), made in Mulgrave, Victoria, Australia. The results are expressed in mg of the mineral element per 100 g of fw. The wavelengths selected for each element were the following: 317.933 nm for Ca determination, 324.754 nm for Cu determination, 238.204 nm for Fe, 769.897 nm for K, 285.213 nm for Mg, 257.610 nm for Mn, 589,592 nm for Na, 177,434 nm for P, 196.026 nm for Se, 213.857 nm for Zn, 267.716 nm for Cr, 184.887 for Hg, and 405.781 nm for Pb determination.

### 4.3. Minor Analytical Determinations: pH, Soluble Solids Content and Total Acidity

The minor analytical determinations were carried out in an aqueous extract with 5 g of fruit (peel and pulp) plus 15 mL of distilled water, which was crushed with a domestic blender to obtain a juice-like substance. 

The pH determination was made by direct potentiometric measurement (AOAC 981.12) of the homogenized fruit juice with pH & Ion-metro GLP 22 Crison (Barcelona, Spain). The determination of the soluble solids content (SSC, °Brix) present in the fruits was carried out by refractometric techniques (AOAC 932.12). The equipment used in this determination was a hand-held refractometer with a range of 0–32 °Brix. Total acidity was determinate potentiometric titration with an alkaline solution (0.5 N NaOH) up to pH = 8.1 (AOAC 942.15) with a Titrino 702 (Metrohm, Herisau, Switzerland) using a Metrohm 6.0420.100 combined Pt selective electrode; the results are expressed in grams of malic acid for 100 g of the fresh sample [89].

### 4.4. Sugars Analytical Determinations: Total Sugars and Glucose

The total sugar content of the samples was obtained using anthrone colorimetric method [90]. The absorbance was read at 625 nm by spectrophotometer (Schott UV line 9400, Mainz, Germany). The results are expressed in g·100 g^−1^ fw.

A glucose oxidase kit was used to determine the D-glucose content [91]. Total sugars were hydrolyzed and decomposed into monosaccharide glucose, which was determined enzymatically by forming a colored compound. The absorbance was read at 540 nm with a spectrophotometer (Schott UV line 9400, Mainz, Germany). The results are expressed in g·100 g^−1^ fw.

### 4.5. Bioactive Compounds Analytical Determinations: Total Phenolic Content and Antioxidant Activity by DPPH Assay

The set of phenolic compounds present in *S. domestica* fruits is oxidized by the Folin-Ciocalteu reagent. Under this foundation, total polyphenols were determined in an aliquot of a methanolic extract by a modification of the Folin–Ciocalteu assay, according to a previously published protocol [92]. The absorbance at 750 nm was measured by spectrophotometer (Jenway 6715/UV-V, Vernon Hills, IL, USA). The results are expressed in mg of caffeic acid for 100 g of *S. domestica* fresh (mg ECA 100 g^−1^ fw). Caffeic acid–Folin–Ciocalteu complex standards were prepared starting from a standard solution of caffeic acid (1000 g L^−1^) (Sigma Aldrich, Shanghai, China). The standards were prepared in a range of 0–10 ppm. The curve equation obtained was Abs = 0.0867 ppm + 0.0075 (R^2^ = 0.9985).

Total antioxidant activity was carried out according to the modified method based on the capture of the free radical DPPH [93,94]. Hydroalcoholic extracts of the sample were used to measure the absorbance at 515 nm wavelength in a UV/V spectrophotometer Jenway 6715/UV-V (Vernon Hills, IL, USA), extrapolating the result in the DPPH-absorbance curve and expressed as µmol equivalent Trolox (TE) per 1 g of fresh *S. domestica* weight (µmol TE·g^−1^ fw).

### 4.6. Analysis of Volatiles Profile

The profile of volatile components was carried out on the whole fruit, including skin and pulp of the fruit since they are the edible fraction of the fruit, at the three stages of ripening: ImF, IF, and RF. Red Delicious apples and quince jelly, both from conventional markets, were used for comparison of volatile composition.

Headspace/solid-phase microextraction (HS/SPME) was used for the extractions of the volatile fraction according to the methodology of Moreno et al. [95]. The analysis of volatiles of the *S. domestica* fruits was performed by 6890 N Network gas chromatography and mass spectrometry (GC-MS), networked to a 5973 inert Mass Selective Detector (Agilent Technologies; Santa Clara, CA, USA). The analytical conditions were as follows: stationary phase silica capillary column (30 m × 0.25 mm i.d. × 0.25 µm thickness film; 5% fenyl-95% methylpolysiloxane); helium carried gas at a constant flow of 1 mL·min^−1^; temperature was maintained at 250 °C. Volatile determinations were performed for each ripening stage of two types of fruit (apple- and pear-shaped) and each reference product (apple and quince jelly). The choice of reference was random, taking a fresh fruit of similar appearance (apple) and a fruit texture similar to that of the fruit in a state of overripeness.

Compounds whose mass spectrum showed a height of more than 80% similarity with the NIST data or according to available standards were considered as identified. For the identification of volatile compounds, a comparison of their mass spectra and GC retention time with reference commercial standards was carried out (RS. Sigma-Aldrich, Saint Louis, MO, USA), or the mass spectrum (MS) was tentatively compared with the Mass Spectral Library (MS Search 2.0) from National Institute of Standards and Technology (NIST) according to other bibliographic data or data from our own research library. For quantification, a total ion current chromatogram (TIC) was employed to integrate the peak area of each compound, similar to other previous works [96]. 

### 4.7. Data Analysis

Three replicates were used to obtain the mean values for nutritional typing, other chemicals, and bioactive components. Datasets from apple and pear shape types were processed using Statgraphics Plus version 5.1 (Manugistics Inc., Rockville, MD, USA) for means, standard errors, and correlations. The analysis of variance (ANOVA; at a significant level of *p* < 0.05) was performed according to a completely randomized design. Differences between types were identified with the F-test and the Kruskal–Wallis test to determine the relationship between the various qualitative parameters. In addition to a description of the aromatic fraction of the different studied fruits, a factorial study was carried out to determine the levels of volatile compounds and the content of each one.

## 5. Conclusions

*S. domestica* species are unknown and undervalued in different areas of the Iberian Peninsula. Knowledge about the nutritional and aromatic fruit value of this underutilized tree should lead to its better protection and dissemination, as well as to its more frequent use in the human diet as a valuable nutrient, mineral, and antioxidant source.

The polymorphism of *S. domestica* fruits generates differentiation in nutritional value. Apple-shaped *S. domestica* fruits show higher values for fat content (although overall fat levels are low), total sugars, glucose, total acidity, Fe, Zn content, and antioxidant activity by DPPH assay than pear-shaped fruits. The two types of *S. domestica* fruits are valued as a good source of potassium, available carbohydrates, and dietary fiber, providing an adequate energy value for a wide range of diets. The intake of these fruits can contribute to the adoption of a healthy dietary pattern that will considerably reduce the footprints of the current diet.

Fruits, in addition to being nutritionally adequate, must be appealing from a sensory point of view. To reach this point of acceptance for consumption, the organoleptic attributes that fruits must meet are a specific texture, a pleasant flavor, and an aroma that invites tasting. Fruit aroma is a key contributor to fruit quality and acceptance by humans. *S. domestica*’s fruit aroma consists of a diverse chemical family, and the presence or absence of certain compounds determines differences in aromas among apple-shaped and pear-shaped fruits.

*S. domestica* fruits have proven to be very original in the aromatic fraction. The fruity aroma is the characteristic aromatic note shared by the several volatile compounds detected. Gas chromatographyw was used to analyze the aroma and revealed that 1-hexanol, hexyl 1,3-octanediol, phenylacetaldehyde, nonanal, hexanal, and α-farnesene were the most potent odor compounds in the overripening stage of the fruits. The aroma profiles of immature *S. domestica* fruits were dominated by aldehydes, while in the overripe stage, the fruit accumulated abundant esters, alcohols, and sesquiterpenoids. The volatile fraction of quince jam (used as control) is different from that of *S. domestica* fruits, and they share some aromatic groups with fresh Red Delicious apple fruits. It would be interesting to investigate whether *S. domestica* fruit shares aromatic groups with other fruits.

*S. domestica* fruits could be introduced as an alternative to seasonal fruit consumption and generate sustainable production and consumption alternatives while recovering cultural and food heritage.

## Figures and Tables

**Table 1:** Individual weights and proximal and mineral parameters of two types of *S. domestica* fruits: mean value ± standard deviation (SD), coefficient of variability (CV), and *p*-value.

Parameter	*S. domestica* Apple-Shaped Type		*S. domestica* Pear-Shaped Type		*p*-Value
	Mean Value ± SD	CV (%)	Mean Value ± SD	CV (%)	
Weight (g)	7.61 ± 0.39	5.15	4.42 ± 0.54	12.23	0.0012 *
Moisture (%)	65.46 ± 0.31	0.47	65.63 ± 1.19	1.81	0.8189
Dry matter (%)	34.54 ± 0.31	0.90	34.37 ± 1.19	3.46	0.8189
Ash (%)	0.51 ± 0.04	7.07	0.60 ± 0.06	9.53	0.0897
Fiber (%)	4.91 ± 1.51	30.79	2.52 ± 0.11	4.29	0.0525
Fat (%)	0.67 ± 0.07	10.45	0.43 ± 0.09	21.44	0.0244 *
Protein (%)	0.54 ± 0.06	10.51	0.68 ± 0.12	18.13	0.1484
Carbohydrates (%)	27.91 ± 1.08	3.88	30.13 ± 1.31	4.33	0.0858
Total energy (kcal 100 g^−1^ fw)	129.65 ± 2.21	1.71	132.20 ± 4.06	3.07	0.3933
Na (mg 100 g^−1^ fw)	7.35 ± 0.14	1.95	6.85 ± 2.21	10.53	0.3100
K (mg 100 g^−1^ fw)	185.56 ± 7.75	4.18	221.19 ± 7.76	3.51	0.0049 *
P (mg 100 g^−1^ fw)	18.16 ± 4.10	22.59	17.19 ± 4.69	27.27	0.8006
Mg (mg 100 g^−1^ fw)	9.81 ± 0.09	0.92	11.12 ± 0.46	4.11	0.0082 *
Ca (mg 100 g^−1^ fw)	44.59 ± 1.84	4.13	43.93 ± 3.58	8.14	0.7894
Mn (mg 100 g^−1^ fw)	0.12 ± 0.10	85.09	0.09 ± 0.01	15.25	0.5740
Fe (mg 100 g^−1^ fw)	1.42 ± 0.23	16.45	0.47 ± 0.10	20.68	0.0029 *
Cu (mg 100 g^−1^ fw)	0.08 ± 0.01	11.52	0.06 ± 0.00	5.72	0.0373 *
Zn (mg 100 g^−1^ fw)	0.11 ± 0.01	12.49	0.06 ± 0.01	13.87	0.0069 *

* Since the *p*-value of the F-test is less than 0.05, there is a statistically significant difference between the mean.

**Table 2:** pH, soluble solids content, total acidity, total sugars, and glucose composition of two types of *S. domestica* fruits: mean value ± standard deviation (SD), coefficient of variability (CV), and *p*-value.

Parameter	*S. domestica* Apple-Shaped Type		*S. domestica* Pear-Shaped Type		*p*-Value
	Mean Value ± SD	CV (%)	Mean Value ± SD	CV (%)	
pH	3.56 ± 0.03	0.81	3.98 ± 0.01	0.25	0.0000 *
Soluble solids content (°Brix)	16.57 ± 0.48	2.90	16.29 ± 10.70	1.81	0.7994
Total acidity (g malic acid 100 g^−1^ fw)	1.17 ± 0.03	2.33	0.79 ± 0.01	1.38	0.0000 *
Total sugars (g·100 g^−1^ fw)	14.19 ± 0.55	3.88	11.42 ± 0.49	4.33	0.0029 *
Glucose (g·100 g^−1^ fw)	6.42 ± 0.25	3.88	5.41 ± 0.17	3.18	0.0044 *

* Since the *p*-value of the F-test is less than 0.05, there is a statistically significant difference between the mean.

**Table 3:** Total phenolic content and antioxidant activity by DPPH assay of *S. domestica* fruits for the two types: mean value ± standard deviation (SD), coefficient of variability (CV), and *p*-value.

Parameter	*S. domestica* Apple-Shaped Type		*S. domestica* Pear-Shaped Type		*p*-Value
	Mean Value ± SD	CV (%)	Mean Value ± SD	CV (%)	
Total phenolics (mg ECA 100 g^−1^ fw)	93.67 ± 19.99	21.34	107.40 ± 7.32	6.81	0.3265
Antioxidant activity by DPPH assay (µmol TE·g^−1^ fw).	283.67 ± 66.16	23.32	170.58 ± 42.08	24.67	0.0669

**Table 4:** Mean value ± standard deviation of esters, sesquiterpenes, sulfur derivative, monoterpenes and alcohols volatile component of *S. domestica* fruits for the two types and three states of maturity: immature (ImF), intermediate (IF) and ripe fruits (RF), and control (apple fruit and quince jam).

Compounds * and Odor Descriptor	*S. domestica* Apple-Shaped Type			*S. domestica* Pear-Shaped Type			Apple Fruit	Quince Jam
	ImF	IF	RF	ImF	IF	RF		
Esters								
Bytil acetate (sweet, fruity)	1.14 ± 1.07	0.33 ± 0.11	0.25 ± 0.05	0.12 ± 0.00	0.12 ± 0.02	0.39 ± 0.18	1.59 ± 0.61	-
Methyl hexanoate (pineapple)	0.21 ± 0.02	-	-	0.33 ± 0.06	0.29 ± 0.04	-	0.14 ± 0.10	-
Hexyl acetate (fruity)	0.47 ± 0.12	0.82 ± 0.26	1.68 ± 0.30	0.47 ± 0.13	0.50 ± 0.06	0.87 ± 0.39	71.68 ± 5.71	-
Methyl octanoate (fruity, floral)	0.13 ± 0.09	0.18 ± 0.13	0.22 ± 0.16	0.19 ± 0.09	0.20 ± 0.02	-	-	-
Ethyl octanoate (fruity, floral)	-	0.13 ± 0.10	0.40 ± 0.17	-	0.11 ± 0.08	0.29 ± 0.04	-	-
Methyl nonanoate (sweet, coconut-like)	-	0.05 ± 0.04	-	0.07 ± 0.00	0.14 ± 0.01	0.12 ± 0.09	-	-
Ethyl nonanoate (fruity brandy-like)	-	-	-	-	-	0.28 ± 0.03	-	-
Sesquiterpenes								
β-caryophyllene (spicy)	-	0.31 ± 0.06	-	-	-	-	-	-
α-farnesene (floral)	4.13 ± 1.38	2.40 ± 0.48	2.84 ± 0.02	5.04 ± 0.41	1.39 ± 0.22	2.47 ± 0.11	-	0.64 ± 0.19
δ-cadinene (woody)	0.05 ± 0.04	0.04 ± 0.03	-	-	-	-	0.28 ± 0.02	-
Sulfur derivative compounds								
Diallyl disulfide (garlic)	0.28 ± 0.04	0.16 ± 0.12	-	0.81 ± 0.31	0.30 ± 0.08	-	-	-
Monoterpenes								
Linalool (floral, fresh, slightly sweet)	0.26 ± 0.19	0.35 ± 0.15	-	0.52 ± 0.22	0.35 ± 0.17	-	-	-
Epoxy linalool (floral, fresh, slightly sweet)	0.18 ± 0.14	0.10 ± 0.09	-	-	1.10 ± 0.06	1.35 ± 0.01	-	-
β-citronellol (smell citrussy)	0.14 ± 0.10	0.16 ± 0.12	-	0.24 ± 0.05	0.13 ± 0.01	-	-	-
Alcohols								
3-metyl-1-butanol (spicy)	-	1.42 ± 1.05	3.18 ± 0.30	0.92 ± 0.09	1.68 ± 0.09	2.61 ± 0.25	-	13.25 ± 0.11
2-methyl-1-butanol (acidic, sharp, spicy)	-	1.53 ± 1.14	4.03 ± 0.09	0.99 ± 0.10	1.51 ± 0.13	2.57 ± 0.87	8.60 ± 1.76	5.58 ± 0.04
1-pentanol (like apricot)	1.39 ± 0.62	0.57 ± 0.13	0.55 ± 0.07	1.09 ± 0.02	1.03 ± 0.10	0.77 ± 0.18	-	-
*cis*-3-hexenol (grass)	0.22 ± 0.16	0.13 ± 0.10	0.05 ± 0.04	0.37 ± 0.08	0.27 ± 0.05	-	-	-
1-hexanol (green, fresh fruit)	35.31 ± 2.00	28.13 ± 0.97	26.20 ± 0.90	25.38 ± 0.25	21.88 ± 0.29	18.94 ± 1.07	15.13 ± 3.74	1.09 ± 0.03
1-heptanol (green)	0.38 ± 0.11	0.26 ± 0.10	0.56 ± 0.14	0.25 ± 0.01	0.38 ± 0.00	0.45 ± 0.04	-	-
6-metil-5-hepten-2-ol (fruity, citrus-like)	0.19 ± 0.14	0.21 ± 0.16	-	0.24 ± 0.03	0.15 ± 0.01	-	-	-
3-octanol (nutty, herbaceous)	0.31 ± 0.23	0.35 ± 0.10	-	0.50 ± 0.02	0.24 ± 0.01	-	-	-
Benzyl alcohol (floral)	-	0.10 ± 0.07	0.50 ± 0.06	-	0.30 ± 0.01	1.99 ± 0.91	-	4.69 ± 0.11
1-octanol (rose, mushroom)	3.38 ± 0.90	2.66 ± 0.60	3.65 ± 0.58	3.34 ± 0.19	1.92 ± 0.03	2.07 ± 0.02	-	
Phenethyl alcohol (floral)	1.30 ± 0.15	2.29 ± 1.70	15.17 ± 2.79	0.90 ± 0.04	2.61 ± 0.07	6.33 ± 1.73	-	13.94 ± 0.27
1,3-octanediol (green, fatty, mushroom)	15.66 ± 2.58	11.24 ± 1.98	15.40 ± 0.59	4.35 ± 1.43	8.30 ± 0.41	11.46 ± 1.23	-	-

* Compounds percentage of total area of identified compounds. “-”: Not detected.

**Table 5:** Mean value ± standard deviation of aldehydes, ketones and methyl ketones, carotenoids, aliphatic and aromatic hydrocarbons, alkanes, carboxylic acids, heterocycles, and other volatile components of *S. domestica* fruits for the two types and three states of maturity: immature (ImF), intermediate (IF) and ripe fruits (RF), and control (apple fruit and quince jam).

Compounds * and Odor Descriptor	*S. domestica* Apple-Shaped Type			*S. domestica* Pear-Shaped Type			Apple Fruit	Quince Jam
	ImF	IF	RF	ImF	IF	RF		
Aldehydes								
Hexanal (green grass)	1.14 ± 0.31	0.97 ± 0.28	0.61 ± 0.08	8.07 ± 1.59	2.21 ± 0.29	1.16 ± 0.03	0.21 ± 0.16	2.83 ± 0.01
2-hexenal (green, fresh fruit)	0.18 ± 0.14	-	-	1.64 ± 0.35	0.06 ± 0.04	-	1.07 ± 0.21	-
Heptanal (strong fruity)	0.11 ± 0.08	0.05 ± 0.03	-	0.09 ± 0.01	0.08 ± 0.00	-	-	0.62 ± 0.00
*cis*-2-heptenal (green)	0.36 ± 0.27	0.50 ± 0.37	-	0.16 ± 0.01	2.76 ± 1.99	-	-	19.89 ± 0.14
Benzaldehyde (fruity, almond-like)	1.78 ± 0.06	2.44 ± 0.38	1.11 ± 0.05	3.37 ± 0.58	2.94 ± 2.09	6.82 ± 0.58	-	3.79 ± 0.11
Phenylacetaldehyde (green floral)	8.67 ± 1.02	8.38 ± 0.86	1.10 ± 0.04	8.65 ± 0.92	12.61 ± 0.50	8.55 ± 0.23	-	-
(E)-2-octenal (fatty, citric)	0.74 ± 0.19	0.77 ± 0.10	0.77 ± 0.04	0.40 ± 0.01	5.56 ± 0.03	0.61 ± 0.02	-	14.12 ± 0.98
Nonanal (fatty, citric)	2.86 ± 0.05	2.23 ± 0.18	2.23 ± 0.58	3.83 ± 0.81	5.21 ± 0.22	4.72 ± 0.16	-	5.66 ± 0.73
Decanal (citric)	0.24 ± 0.03	0.17 ± 0.03		0.16 ± 0.00	0.20 ± 0.01	0.08 ± 0.06	-	-
Ketones/methyl ketones								
2-heptanone (fruity, banana-like)	0.27 ± 0.20	-	0.90 ± 0.26	-	0.10 ± 0.07	0.21 ± 0.05	-	-
Acetophenone (sweet, floral, citric)	0.04 ± 0.03	-	0.15 ± 0.03	-	-	-	-	-
Carotenoids								
6-metil-5-hepten-2-ona (fruity)	0.27 ± 0.20	0.37 ± 0.28	-	1.81 ± 0.02	1.18 ± 0.27	-	-	6.83 ± 0.07
Aliphatic and aromatic hydrocarbons								
β-methylnaphthalene (floral)	0.66 ± 0.06	0.21 ± 0.18	0.62 ± 0.44	0.17 ± 0.01	0.16 ± 0.11	-	-	-
α-methylnaphthalene (coal tar)	0.20 ± 0.06	0.06 ± 0.04	-	0.08 ± 0.01	-	-	-	-
Alkanes								
Tetradecane (gasoline-like)	0.09 ± 0.07	0.08 ± 0.06	0.15 ± 0.00	0.07 ± 0.00	-	-	-	-
Carboxylic acids								
Hexanoic acid (barnyard animals)	0.46 ± 0.34	0.45 ± 0.34	-	0.23 ± 0.16	-	-	-	-
Nonanoic acid (rancid)	-	-	0.73 ± 0.10	0.63 ± 0.04	0.41 ± 0.04	0.43 ± 0.31	0.05 ± 0.04	-
Lauric acid (bay, coconut)	0.85 ± 0.48	0.62 ± 0.03	2.07 ± 0.29	0.72 ± 0.32	0.81 ± 0.12	2.20 ± 1.05	1.24 ± 0.38	7.08 ± 0.77
Heterocycles								
γ-hexalactone (herbal)	-	0.10 ± 0.08	0.13 ± 0.09	0.17 ± 0.02	0.14 ± 0.00	0.09 ± 0.06	-	-
γ-nonalactone (creamy coconut)	0.16 ± 0.01	0.14 ± 0.01	0.09 ± 0.07	0.15 ± 0.02	0.15 ± 0.01	0.14 ± 0.10	-	-
γ-decalactone (fruity)	0.66 ± 0.09	0.50 ± 0.07	0.68 ± 0.07	0.25 ± 0.02	0.29 ± 0.02	0.39 ± 0.04	-	-
Others								
2,4,5-trimethyl-1,3-dioxolane (nutty, phenolic)	13.84 ± 0.67	25.50 ± 5.04	10.75 ± 1.91	22.41 ± 1.01	24.04 ± 0.57	20.13 ± 0.10	-	-
2-pentylfuran (fruity, green)	0.21 ± 0.15	0.24 ± 0.10	-	0.17 ± 0.00	0.28 ± 0.03	0.20 ± 0.14	-	-
Rose oxid (green, floral, rose)	0.17 ± 0.12	0.17 ± 0.12	-	0.28 ± 0.03	0.19 ± 0.02	0.11 ± 0.08	-	-
Camphor (fatty)	0.92 ± 0.10	2.13 ± 0.14	3.27 ± 0.04	0.67 ± 0.11	0.72 ± 0.11	1.22 ± 0.20	-	-

* Compounds percentage of total area of identified compounds. “-”: Not detected.

**Table 6:** Mean value ± standard deviation (compounds percentage of total area) for chemical families of *S. domestica* fruits in the two types and three states of maturity: immature (ImF), intermediate (IF) and ripe fruits (RF), and control (apple fruit and quince jam). “-”: Not detected.

Chemical Family	*S. domestica* Apple-Shaped Type			*S. domestica* Pear-Shaped Type			Apple Fruit	Quince Jam
	ImF	IF	RF	ImF	IF	RF		
Esters	1.68 ± 0.86	2.22 ± 0.24	2.93 ± 0.66	1.19 ± 0.01	1.41 ± 0.15	1.96 ± 0.43	71.83 ± 5.02	-
Sesquiterpenes	3.65 ± 1.35	2.99 ± 0.51	3.04 ± 0.02	4.97 ± 0.41	1.39 ± 0.22	2.47 ± 0.11	0.29 ± 0.02	0.64 ± 0.19
Sulfur derivative	0.28 ± 0.04	0.16 ± 0.01	-	0.81 ± 0.03	0.30 ± 0.08	-	-	-
Monoterpenes	0.89 ± 0.23	0.94 ± 0.18	-	1.26 ± 0.54	1.58 ± 0.13	1.34 ± 0.01	-	-
Alcohols	57.52 ± 0.65	62.57 ± 2.78	74.25 ± 2.41	38.24 ± 0.79	40.18 ± 0.13	46.75 ± 1.81	25.32 ± 5.50	38.54 ± 0.29
Aldehydes	16.81 ± 1.97	19.26 ± 1.40	6.12 ± 0.79	26.36 ± 0.90	26.60 ± 0.55	21.92 ± 0.87	1.37 ± 0.11	46.90 ± 0.22
Ketones/methyl-ketones	0.55 ± 0.23	-	1.16 ± 0.29	-	0.16 ± 0.07	0.21 ± 0.05	-	-
Carotenoids	0.27 ± 0.20	0.37 ± 0.28	-	1.81 ± 0.02	1.18 ± 0.27	-	-	6.83 ± 0.07
Aliphatic and aromatic hydrocarbons	0.80 ± 0.12	0.36 ± 0.15	0.92 ± 0.44	0.26 ± 0.05	0.24 ± 0.11	-	-	-
Alkenes	0.09 ± 0.07	0.08 ± 0.06	0.15 ± 0.00	0.07 ± 0.00	-	-	-	-
Carboxylic acids	1.47 ± 0.25	1.71 ± 0.31	2.94 ± 0.38	1.73 ± 0.48	1.22 ± 0.08	2.95 ± 1.32	1.18 ± 0.42	7.09 ± 0.77
Heterocycles	0.77 ± 0.08	0.94 ± 0.02	1.10 ± 0.11	0.57 ± 0.06	0.58 ± 0.01	0.74 ± 0.13	-	-
Others	14.94 ± 0.31	7.67 ± 4.72	7.38 ± 0.88	23.29 ± 1.15	25.17 ± 0.67	21.66 ± 0.21	-	-

## Data Availability

The data presented in this study are available on request from the corresponding author due to not having a repository, at the moment.
